# Alternative strategies in the acquisition of home ranges by male pine martens in a high-density population

**DOI:** 10.1007/s13364-012-0086-9

**Published:** 2012-07-06

**Authors:** Andrzej Zalewski

**Affiliations:** Mammal Research Institute, Polish Academy of Sciences, 17-230 Białowieża, Poland

**Keywords:** *Martes martes*, Reproductive strategies, Spacing pattern, Solitary carnivores, Optimality theory

## Abstract

Two strategies of home range acquisition by male subadult pine martens (*Martes martes*) were described from a high-density population inhabiting Białowieża National Park. Four mother–offspring pairs were identified by genetic parentage assignments. Four subadult males showed two different strategies of home range acquisition: dispersal and sedentary. The dispersing males used an area 4–10 times larger than in sedentary subadult males. A sedentary subadult male used his natal area with his mother, and in the following mating season, this male left this area and established a home range that overlapped greatly with another unrelated female near the natal range. A similar high overlap between another subadult male and an unrelated adult female persisted for 3 years. After the death of this female, the male extended his range to overlap slightly with two to four other females. The sedentary strategy adopted by some subadult males may explain the great variation in spacing patterns of solitary mammals.

Maximizing reproductive success in males is mainly related to the number of females that males encounter in polygynous and promiscuous mating systems (Clutton-Brock 1988). In solitary species, males select their home range based on the spatial distribution and density of females (Sandell 1989). The lifetime reproductive success of males is also related to the number of seasons in which they reproduce. Therefore, the best strategy for males is to start reproducing early in order to increase the number of litters they sire during their lifetime. In territorial species this is related to the acquisition of an area overlapping with many females. For subadult males, one strategy to acquire a territory is to disperse, often over a long distance, find a free territory and then reproduce. During dispersal, individuals may occasionally fertilize females en route (e.g. Reichard et al. 2004). However, this strategy carries a high mortality risk (Byrom and Krebs 1999; Waser and Jones 1983) connected with travelling for long distances across unknown and often unfavourable habitats. Another strategy is to stay in the vicinity of their natal range in a known area and wait for the chance to obtain a territory after the death of neighbouring individuals. In populations with high densities of mature males and low individual turnover, the chances that a subadult male obtains a territory in their first year are low (Costello et al. 2009). Therefore, in order to optimise their reproductive chances, subadult males should select different reproductive strategies in relation to their individual characteristics (e.g. body size), or the mortality risk, operational sex ratio or density of adult males in the environment (Clutton-Brock 1988; Costello et al. 2009).

Mustelids employ a promiscuous or polygynous mating system and commonly show intrasexual territoriality, where male territories exclude other males and female territories exclude other females, but male territories overlap with those of females (Powell 1979; Sandell 1989). Similar to other solitary mammals, adult male mustelids use two main strategies to increase reproductive success: staying and guarding females within a territory or roaming to find receptive females (Sandell 1989; Costello et al. 2009). Therefore, in most mustelids, spacing patterns are not stable between consecutive years, especially in *Mustela* species like weasels (*Mustela nivalis*) and stoats (*Mustela erminea*), that live only for 2–3 years (King 1989). Marten species show different spacing patterns. In contrast to *Mustela* species, martens show very high fidelity to their ranges, and small seasonal and between-year variation in range size that stabilizes spacing patterns and population dynamics (O'Doherty et al. 1997; Zalewski and Jędrzejewski 2006). Furthermore, the annual mortality of martens is much lower than in other mustelids of similar body size (Caley and Morriss 2001; Bartoszewicz and Zalewski 2003; Zalewski and Jędrzejewski 2006; McCann et al. 2010). Very high survival and site fidelity of female martens favour males that stay and maintain exclusive access to females. In such situations, the possibility that subadult individuals find empty areas for territory acquisition is relatively low. How do subadult male martens acquire their own territories in populations with high densities and stable spacing patterns? In this paper, I used genetic parentage assignments and radiotracking data of pine marten to analyse two possible strategies utilized by subadult male to acquire a territory in a high-density population inhabiting a deciduous forest. I also discuss how selection of a particular strategy affects the spacing pattern of martens.

The study area was conducted in northeastern Poland (52°43′ N, 23°54′ E) in the Białowieża National Park (47.5 km^2^) which is part of a large primeval woodland covering over 1,250 km^2^. The old-growth forest of Białowieża National Park is dominated by oak–lime–hornbeam stands (44 % of the area) consisting of hornbeam *Carpinus betulus*, oak *Quercus robur*, lime *Tilia cordata*, maple *Acer platanoides* as well as a small amount of Norway spruce *Picea abies*. The two other main forest types are mixed coniferous dominated by Norway spruce and pine *Pinus silvestris* and ash–alder forests dominated by black alder *Alnus glutinosa* and ash *Fraxinus excelsior*. The study area has not been exploited for timber, trapping or hunting, and human utilization is very low. The pine marten inhabiting the very rich, pristine woodlands of Białowieża National Park existed at a very high density (almost the highest reported in Europe; Zalewski and Jędrzejewski 2006).

The pine marten was captured using box live traps, baited with chicken eggs and honey. Captured martens were immobilized with an injection of Ketamine hydrochloride (50 mg/ml, Narkamon), weighed, sexed, aged (based on tooth wear) and equipped with radio collars (AVM or Lotek; 12–20 g, less than 2 % of the lightest martens’ body weight; life span 5–7 months). Martens were located once per day or were continuously monitored for 4–24 h on certain days (see Zalewski and Jędrzejewski 2006 for further details on radiotracking). Home range size was estimated using minimum convex polygons applied in the software Tracker ver.1.1. Pooled data were analysed in three seasons: spring (16 March–15 June), summer (16 June–15 October) and autumn–winter (16 October–15 March). More information on the methods, list of other radiotracked adult individuals, their home range sizes and distribution is given in Zalewski and Jędrzejewski (2006).

Tissue samples from 13 pine martens were obtained from Białowieża Forest. Samples were obtained from live-trapped martens (Zalewski and Jędrzejewski 2006) and from individuals found dead between 1991 and 1999. All tissue samples were stored at −20 °C prior to DNA extraction. DNA was extracted from tissue samples using a DNeasy Tissue Kit (Qiagen) according to the manufacturer’s instructions. Nine microsatellite loci were used to genotype individuals: Lut604, Ma1, Ma2, Ma19, Mel10, Gg7, Gg14A, Gg14B and Gg454 (Dallas and Piertney 1998; Davis and Strobeck 1998, Walker et al. 2001). Microsatellites were amplified in four duplex reactions prepared using a Multiplex PCR Kit (Qiagen). Reaction mixtures contained approximately 0.5 μl of template DNA in a total volume of 8.0 μl. The thermal cycle consisted of an initial denaturation step at 95 °C for 15 min, followed by 30 cycles of 94 °C for 30 s, 57 °C for 1 min 30 s and 72 °C for 1 min. Amplified fragments were resolved by electrophoresis using an ABI 3100 Genetic Analyzer (Applied Biosystems) using GeneMapper 3.5 (Applied Biosystems). The size marker used was Gene Scan Rox 400HD (Applied Biosystems). To assign parentage we used a likelihood method within the software COLONY version 2.0.11 (Jones and Wang 2010). The software COLONY was run with the full-likelihood analysis, assuming polygamy without inbreeding for both males and females, and no maximum number of siblings or known relatives. The analyses were run three times to ensure consistent parentage assignments.

From 1991 to 1999, 18 pine martens were captured (4 subadult, 5 adult males, and 9 adult females) in Białowieża National Park and in the surrounding forests. Analyses of genetic relatedness between martens revealed four mother–offspring dyads with a probability higher than 0.85, using the likelihood method: F8-F11, F8-F12, F20-M7 and F11-M14. The other individuals (including two subadults males M5 and M1) were not related to known females. Two females (F11 and F12) established their home ranges 1–2 km from their natal ranges (F8), and one male (M7) established his home range 1 km from his natal range (F20).

Subadult males showed two different strategies of space use: dispersal and sedentary. The dispersing males (M1 and M14) used areas 4 to 10 times larger than in sedentary subadult males (Fig. 1). In spring, M1 dispersed in suboptimal habitat, a park in the village of Białowieża, and once every 1–2 weeks undertook a long foray (4–6 km) to the Białowieża National Park. M14 moved each day for long distances in a circular pattern in the vicinity of his mother’s (F11) home range (Fig. 2). Sedentary subadult male (M7) used the natal area with his mother (F20) in winter 1994/1995 (Fig. 3). In spring 1995, M7 still partly used this natal range but extended his range to overlap with an unrelated female (F12). In summer 1995, M7 moved and reduced the size of his home range so that it overlapped extensively with the home range of F12 (Fig. 3). Similar high overlap was observed between subadult male M5 and an unrelated adult female (F8) in 1992 (Fig. 3). Despite the fact that the home ranges of M5 and F8 overlapped to a large degree, M5 was not the father of her offspring (F12) born in 1992. This high overlap of M5 and F8’s home ranges was observed for the next 3 years (Fig. 4). After the death of F8 in the spring of 1994, M5 extended his home range to overlap a small amount (4 % ± 0.2 SE) with two to four other females in the spring–summer of 1995 (Fig. 3).

**Fig. 1:**
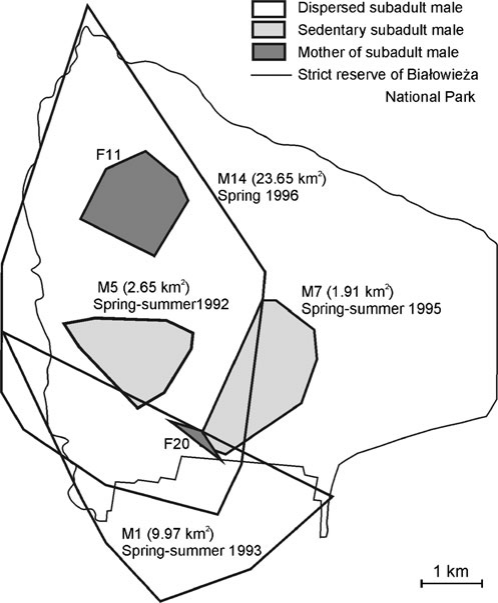
Area used by two dispersing subadult males (M1 and M14) and two sedentary subadult males (M5 and M7) in relation to their mother’s home range (F11 mother of M14; F20 mother of M7). The numbers in parentheses indicated homer range size. The number of locations used in the calculation of home range size: M1—75, M5—290, M7—410, M14—96, F11—316, F20—3. F20 was tracked for only a few days, and thus, her home range is approximately estimated

**Fig. 2:**
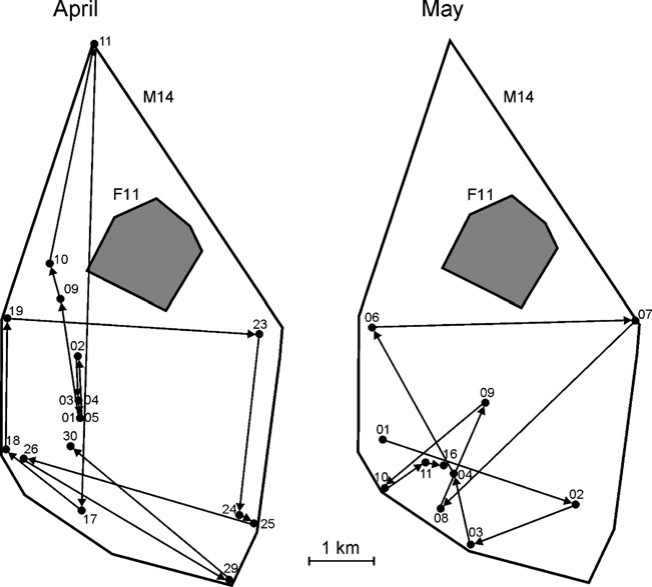
Moving pattern of dispersing subadult male (*M14*) in relation to his mother’s home range (*F11*) during 2 months of the spring of 1996. The *numbers* indicate consecutive days of the month

**Fig. 3:**
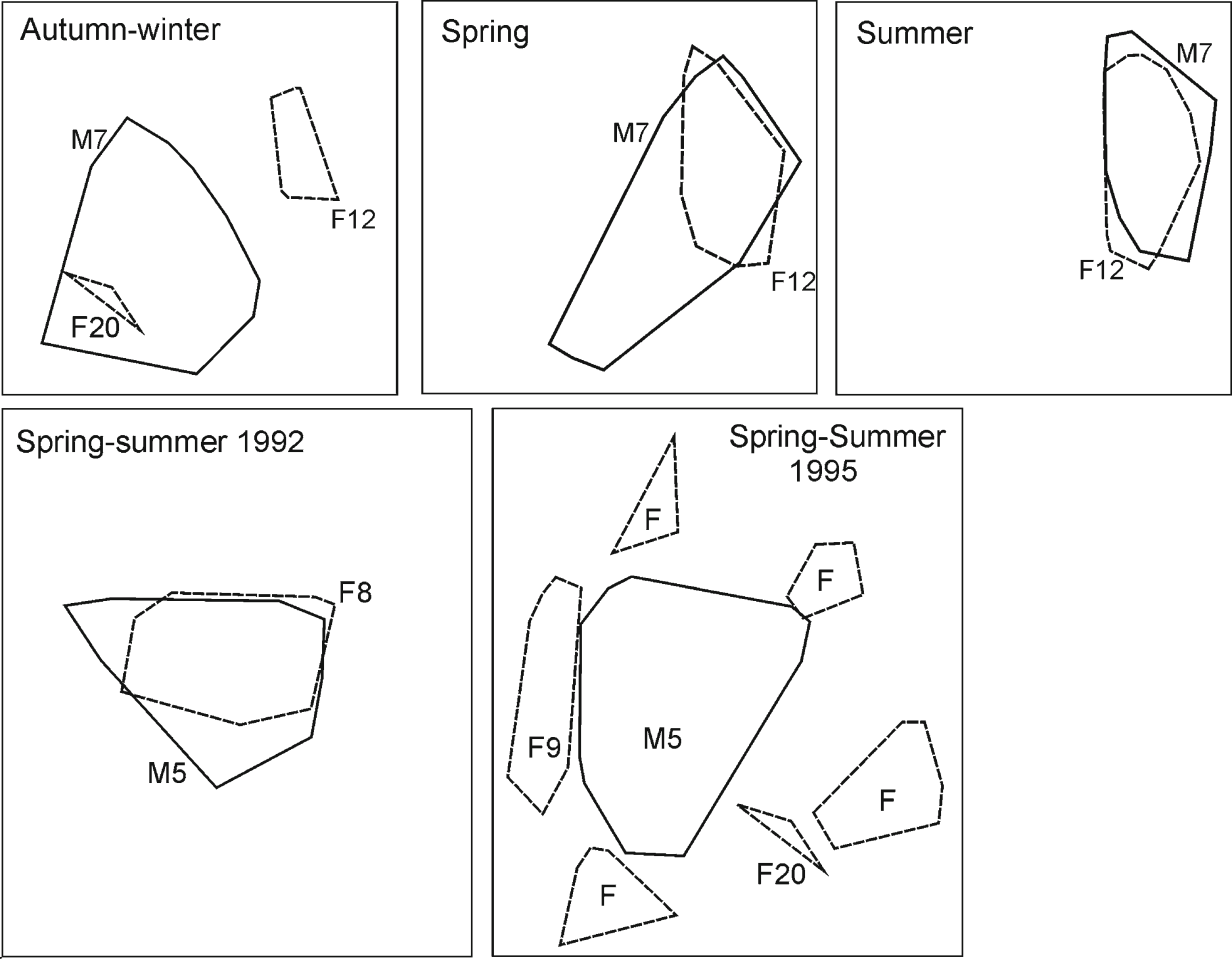
*Upper panel*: shift of the home range of a pine marten subadult male (*M7*) from the natal range (*F20*) to a new range overlapping with one female (*F12*; sedentary strategy). *Lower panel*: extended home range of a sedentary male (*M5*) after the death of a female (*F8*). Male—*solid line*; female—*dashed line*. Female home ranges without numbers were snow tracked (for more detailed information, see Zalewski and Jędrzejewski 2006)

**Fig. 4:**
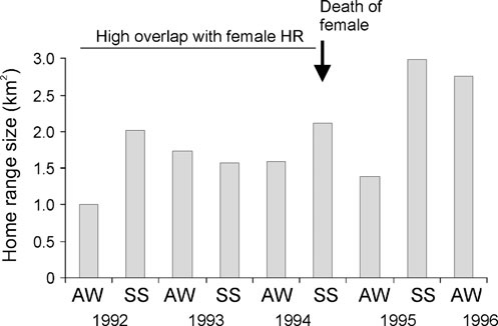
Autumn–winter (*AW*) and spring–summer (*SS*) home range size variation of sedentary male (*M5*) over 5 years in relation to a presence female (*F8*)

These results show two possible strategies of home range acquisition by subadult male pine martens: dispersing to find empty areas or acquiring part of the home range of a non-related female near the natal range. Despite the fact that these observations are based only on four subadult males, the two strategies described here provide a possible explanation for the great variation in degree of home range overlap between males and females in solitary carnivores. The sedentary strategy may occur in populations in which female home ranges overlap a small amount with male home ranges, and neighbouring adult males do not exclude subadult males. In pine marten populations inhabiting Białowieża Forest, intersexual overlap is small (9–27 %; Zalewski and Jędrzejewski 2006). Furthermore, in this population, there is low seasonal variation in home range size (20–25 %) and high fidelity between consecutive years (Zalewski and Jędrzejewski 2006), similar to American martens *Martes americana* (Phillips et al. 1998). In contrast to *Mustela* species (weasels and stoats, Erlinge and Sandell 1986; Sandell 1989) but similar to American martens (Katnik et al. 1994), no roaming behaviour in adult males was observed in this study. All these allow subadult males to settle between adult male home ranges, inside the home range of adult females.

Subadult males which occupied a small home range overlapping with only one female may increase their chances of reproductive success by guarding females. In spring and summer, subadult males used the same resting sites as females and indeed spent some time resting together during breeding season (Zalewski 1997). This should potentially allow the subadult male to reproduce. It is interesting that the male that chose the sedentary strategy and established his home range inside the female home range held onto this range as long as the female lived, and extended his range after her death to increase the overlap with the home ranges of other females. An increase in home range size of both adult and subadult males after a female’s death usually occurs just before the spring–summer mating season, even if the female died at the end of the previous summer or autumn the year before (Zalewski and Jędrzejewski 2006 and this study). In studies of pine marten spacing patterns conducted over 1 or 2 years (e.g. Marchesi 1989; Herr et al. 2009), observations of large range overlap between 2–4-year-old males and adult females are probably a consequence of a sedentary strategy adopted by males when they were subadults. This possibility highlights the need to conduct long-term studies to further document temporal changes in marten spacing patterns.

In solitary carnivore species, patterns of space use have been described as having high intrasexual territoriality but low intersexual territoriality (Powell 1979, 1994; Sandell 1989). However, competition between males and females for food (Zalewski 2007) promotes both sexes to use separate ranges with limited overlap. Intra- and intersexual territorial behaviour provides a mechanism by which local density might be limited and flexibility in this behaviour may affect population dynamics (Newton 1998). Pine marten population dynamics in Białowieża Forest are influenced mainly by social interactions and, to a smaller degree, by food abundance (Zalewski and Jędrzejewski 2006). Flexibility in space partitioning between individuals can exert strong effects on the population dynamics of territorial animals, with this directly influenced by alternative tactics of subadult males to acquire territories.
